# Robustness of radiomics to variations in segmentation methods in multimodal brain MRI

**DOI:** 10.1038/s41598-022-20703-9

**Published:** 2022-10-06

**Authors:** M. G. Poirot, M. W. A. Caan, H. G. Ruhe, A. Bjørnerud, I. Groote, L. Reneman, H. A. Marquering

**Affiliations:** 1 Department of Radiology and Nuclear Medicine, Amsterdam UMC, location University of Amsterdam, L0-148 Meibergdreef 9, 1105 AZ Amsterdam, The Netherlands; 2 Department of Biomedical Engineering and Physics, Amsterdam UMC, location University of Amsterdam, Amsterdam, The Netherlands; 3 Department of Psychiatry, Radboudumc, Nijmegen, The Netherlands; 4 Donders Institute for Brain, Cognition and Behavior, Radboud University, Nijmegen, The Netherlands; 5 Department of Psychology, University of Oslo, Oslo, Norway; 6 Department of Physics, University of Oslo, Oslo, Norway; 7 Division of Radiology and Nuclear Medicine, Computational Radiology and Artificial Intelligence (CRAI), Oslo University Hospital, Oslo, Norway; 8 Department of Radiology, Vestfold Hospital Trust, Tønsberg, Norway

**Keywords:** Predictive markers, Biomarkers, Translational research, Cancer, Computational biology and bioinformatics, Biomarkers, Health care, Medical imaging, Magnetic resonance imaging, Computational neuroscience, Neuroscience

## Abstract

Radiomics in neuroimaging uses fully automatic segmentation to delineate the anatomical areas for which radiomic features are computed. However, differences among these segmentation methods affect radiomic features to an unknown extent. A scan-rescan dataset (n = 46) of T1-weighted and diffusion tensor images was used. Subjects were split into a sleep-deprivation and a control group. Scans were segmented using four segmentation methods from which radiomic features were computed. First, we measured segmentation agreement using the Dice-coefficient. Second, robustness and reproducibility of radiomic features were measured using the intraclass correlation coefficient (ICC). Last, difference in predictive power was assessed using the Friedman-test on performance in a radiomics-based sleep deprivation classification application. Segmentation agreement was generally high (interquartile range = 0.77–0.90) and median feature robustness to segmentation method variation was higher (ICC > 0.7) than scan-rescan reproducibility (ICC 0.3–0.8). However, classification performance differed significantly among segmentation methods (p < 0.001) ranging from 77 to 84%. Accuracy was higher for more recent deep learning-based segmentation methods. Despite high agreement among segmentation methods, subtle differences significantly affected radiomic features and their predictive power. Consequently, the effect of differences in segmentation methods should be taken into account when designing and evaluating radiomics-based research methods.

## Introduction

Radiomics is an established method for quantitative analysis of radiological images. It involves processing of medical images to extract large numbers of quantitative image features^1–3^. In clinical oncology, radiomics has (greatly) contributed to prediction of patient outcome and clinical decision-making support^4^. The radiomics approach has also seen applications in early diagnosis of Alzheimer’s disease using positron emission tomography (PET)^5,6^. These successes have increased interest for applications of radiomics in other disciplines, such as psychiatry. The application of radiomics for psychiatric disorders constitutes to a relatively new field of research: psychoradiology^7–9^.

Radiomics in psychiatry has seen several applications such as classification, prediction, and treatment selection^7,9^ in diseases like schizophrenia^10–14^, attention hyperactivity disorder^15^, bipolar disorder^16^, and major depressive disorder^17,18^. In these applications, various magnetic resonance imaging (MRI) modalities have been employed such as structural T1-weighted imaging, T2-FLAIR-weighted imaging, diffusion tensor imaging (DTI), functional MRI (fMRI), and arterial spin labeling (ASL).

Despite these promising applications, reliability of radiomics has been hindering its broad validity and generalizability. Variability and uncertainty can be introduced in each step in the six-step radiomics pipeline: first, image acquisition^19–27^, processing^26,28–37^ and segmentation^2,3,38–42^, then feature extraction and selection, and finally statistical inference^1,3,38,39,43,44^.

Reliability of radiomics with respect to image acquisition and image processing has been under extensive scientific scrutiny, especially in oncology, where 87% of studies on robustness of radiomic features has been performed^45^. This research has yielded standardization of MRI-acquisition protocols and standardization of radiomics definitions that the field of psychoradiology can draw from^46,47^. However, the segmentation in oncology has mainly been focused on effects of semi-automatic segmentation^37,48–53^ of tumor mass rating, whereas psychoradiology makes use of different fully-automatic whole-brain anatomical segmentation methods^54^. Thus, oncological findings on robustness to segmentation methods are hard to translate to psychoradiology.

Radiomics has also shown promising applications in the domain of PET imaging^55^. Here, robustness of radiomics features to co-registration has been shown to impact outcome prediciton^56^ Radiomic features have also been used in a comparative method to assess the sensitivity and performance of segmentation methods^57,58^. The importance of robustness for prediction has also been researched in PET^56^. These works share the same motivation for ascertaining robustness and provide methods for it. However, the scope of existing literature does not cover our imaging modalities, anatomy of interest, and consequently segmentation methods of use.

Thus, domain specific research focusing on the robustness of brain MRI radiomic features for psychoradiology has been lacking. To our knowledge, only one study has been published in this respect. In this study, Li et al.^59^ found that texture features are the most reproducible brain MRI features in T1-weighted images for hippocampal segmentations. Whole-brain and subcortical segmentation accuracy was assessed in comparative settings by several authors^60–62^ Other studies that investigated segmentation accuracy were limited to one or two anatomical regions, such as the hippocampus^63–68^, amygdala^66^ and caudate and putamen^69^, but have not reported effects on radiomic features. In this work, we analyze the impact of variations in segmentation methods on a full radiomics pipeline that uses DTI and T1-weighted radiomic features for classification purposes. Segmentation methods included are two established methods: FreeSurfer SAMSEG^70,71^ and FreeSurfer ASEG^72^. Two other methods included are recent deep learning-based methods: FastSurfer^61^ and Med-DeepBrain^62^. We analyze this full radiomics pipeline in three subsequent steps:

- We analyzed segmentation agreement between pairs of segmentation methods to aid interpretation of following robustness findings.
- We analyzed robustness of radiomic features to segmentation method variation by computing the ICC across segmentation methods for each radiomic feature. We compare these numbers to scan-rescan reproducibility of radiomic features.
- We compared discriminative power of the radiomic features generated by these four segmentation methods by subjecting them to a diagnostic test, consisting of classifying sleep-deprived subjects and non-deprived controls. Literature has shown subtle differences in changes in structural and diffusion weighted imaging after sleep deprivation^73–77^. For sake of dimensionality reduction and generalizability, classification was performed on a subset of all features, as is recommended in literature^27,78–80^.

## Results

Of all 46 participants, data were present and consistent. No subjects had to be excluded for initial analysis. None of the segmentations nor DTI co-registrations failed. Thirty-two anatomical regions were available for all segmentation methods. Two examples of such segmentations are provided in Supplementary Fig. S1. Subsequently, radiomic feats were extracted from T1-weighted and DTI data over four time points (TPs). The total number of features extracted was 107, each of which can be attributed to one of the seven feature classes mentioned earlier.

### Segmentation agreement

**Figure 1:**
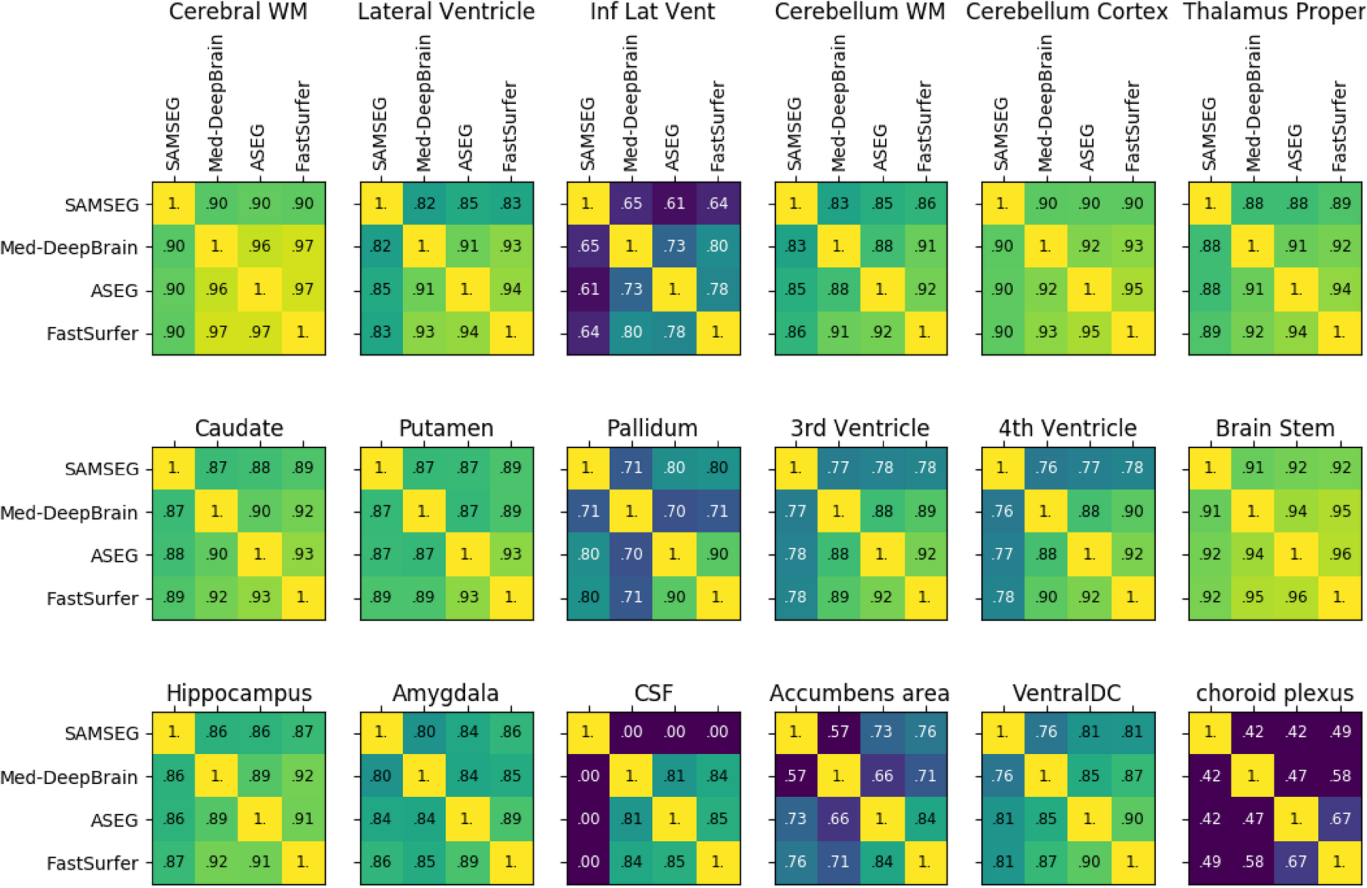
Pairwise segmentation agreement matrix. Dice-coefficients between each pair of segmentation methods for each subcortical area. Left–Right averages are shown where possible. Clarification of abbreviations: *WM* white matter, *DC* diencephalon, *CSF* cerebral spinal fluid, *Inf Lat Vent* inferior lateral ventricle.

Dice-coefficients were computed for each segmentation method pair. Figure 1 shows segmentation agreement for each anatomical area, but left–right averaged where possible. Segmentation agreement as computed for each anatomical area was high (inter quartile range (IQR) = 0.77–0.90). Average agreement was highly correlated (ρ = 0.93) between left and right anatomical areas, with the exception of the pallidum where left scored worse than right with a Dice-score difference of 0.1.

### Radiomic feature reliability

**Figure 2:**
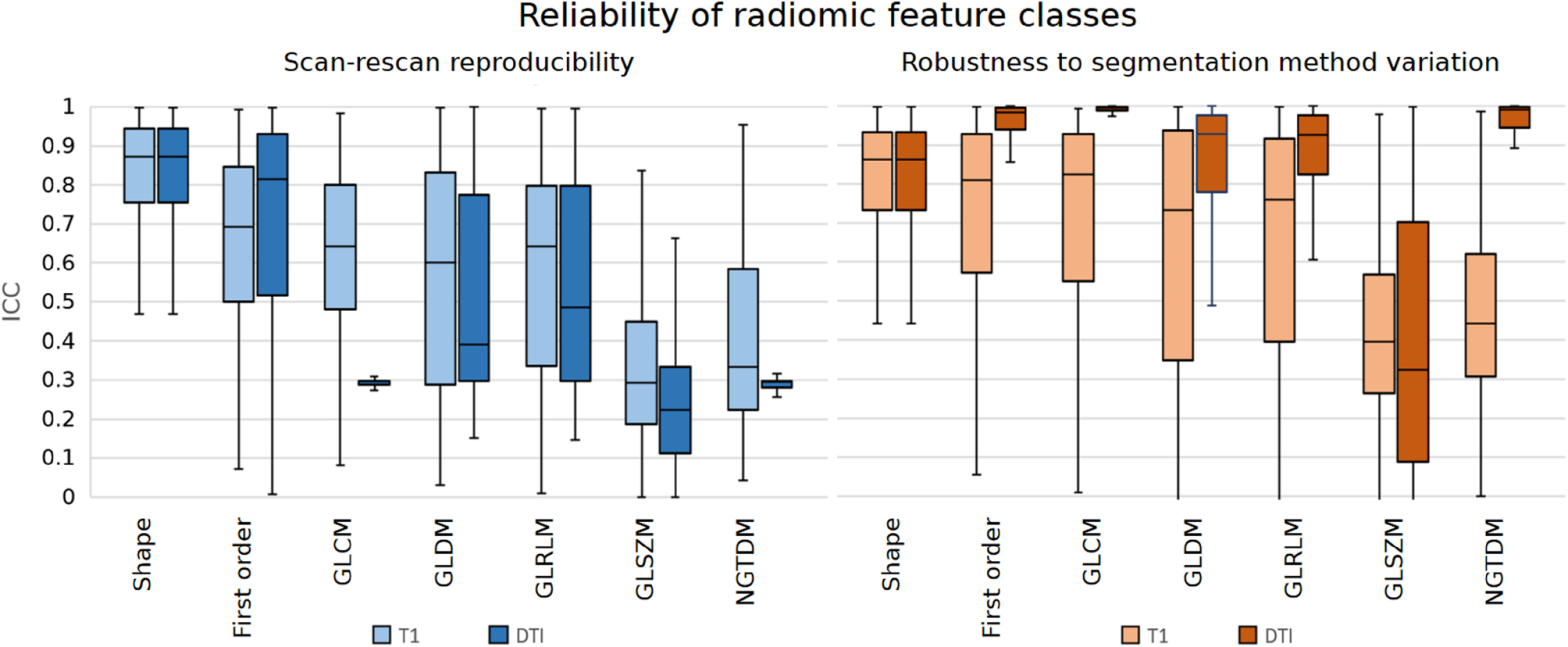
Reproducibility and robustness for each class of radiomic features. For an explanation of abbreviations see Supplementary Note S6.

Supplementary Fig. S2 provides an exhaustive overview of radiomic feature reliability as calculated for each feature, for each anatomical region, for each modality and according to the two earlier defined metrics: scan–rescan reproducibility and robustness to segmentation method variation. Remaining values were averaged over anatomical regions and feature class (Fig. 2). The choroid plexus, inferior lateral ventricles and cerebrospinal fluid (CSF) were excluded due to subpar segmentation quality as prescribed in methods Sect. 4.4.

### Feature selection and classification

For the sleep deprivation classification, four subjects were excluded because of missing data, one of which was in the sleep wake cycle (SWC) group. Thus, the remaining cohort consisted of 22 subjects in the normal SWC group and 20 in the sleep-deprived group. The training partition consisted of 30 subjects, the validation partition of eight subjects, and the test set of four subjects.

Initially, about 27 thousand features were generated for each subject: 107 radiomic features, 32 anatomical regions, four TPs and two modalities. A subset of features was selected for subsequent analysis in three steps described in “Radiomics-based classification and statistical analysis”. In the first step, only first-order shape and first-order-based features of the DTI images were included. In the second, excluded anatomical regions are the choroid plexuses, the third and fourth ventricles and the CSF. After feature selection, only 640 features remained (32 radiomic features, 20 anatomical regions, one coefficient-over-time, one modality). As side analysis, we compared this approach with Chi-Squared based data driven feature selection for which the results can be found in Supplementary Table S1.

Computation time per fold amounted to be about a minute. Median performance results were computed both in terms of the training loss optimized (BCE) as well as classification accuracy (Table 1). The Friedman test yielded 31.18 (p value < 10^–6^), rejecting the hypothesis that performance was similar among different pipelines. Post hoc testing found that performance using ASEG significantly differed from other methods, and FastSurfer differed from SAMSEG. Full results of post hoc testing can be found in Supplementary Table S2.

**Table 1:** Classification performance of pipelines for each segmentation method. Median performance of cross-validation folds and IQR are shown. Best performance shown in bold.

	BCE loss	Accuracy
SAMSEG	0.53 (0.37–0.70)	77% (60–89%)
MED-DEEPBRAIN	0.50 (0.30–0.69)	82% (62–91%)
ASEG	0.60 (0.47–0.72)	71% (62–82%)
FASTSURFER	**0.46 (0.25–0.66)**	**84% (74–92%)**

## Discussion

In this work, we have found that despite high whole-brain segmentation agreement, radiomic feature robustness to variability among these selected segmentation methods is only moderate. Consequently, this variability significantly affects radiomics-based classification performance.

As compared to literature, this work presents three novel analyses. First, this work presents a broader analysis of radiomic feature reliability than previously presented in literature, by including both a wide range of subcortical areas as well as two MRI modalities, i.e., T1- and diffusion weighted MRI.

Second, whereas existing literature is generally confined to one-to-one comparisons of methods, our work provides an independent comparison of four segmentation methods. The unbiased nature of this work counters a potential bias in favor of a newly introduced methods in study design. In addition, by including four segmentation methods in the same setup, this work provides a more comparable comparison among them.

Third, as opposed to most literature concerning comparison of sensitivity of whole-brain segmentation methods, our work takes a radiomic approach. We thereby go beyond other work testing sensitivity on volume measurements alone, such as in application to Alzheimer’s disease^61,62^.

### Segmentation agreement

In the first part of this work, we have analyzed the agreement between segmentation method pairs. Disagreement can stem from a variety of sources. For one, it can be anatomically dependent. Insufficient contrast of the specific anatomy in T1 can make certain anatomical regions hard to segment. In addition, a high surface-to-volume ratio can leave certain anatomical regions vulnerable to rapid reduction of Dice-coefficients. Non-anatomically inherent disagreement can be caused by differences in segmentation labeling definitions. This is the likely reason for the low Dice-coefficients in the inferior lateral ventricle and CSF produced by the SAMSEG method as compared to the other three methods. Additionally, differences in labeling definitions in atlases for conventional methods or training data for deep learning can be the root of systemic bias. This might be the cause for slightly reduced agreement between SAMSEG and the other methods, e.g., for cortical GM and WM. Thus, all methods showed high agreement, with only SAMSEG showing slight deviation.

### Reliability of radiomic features

The second part of this work concentrated on the effects on radiomic feature reliability, broken down into scan-rescan reproducibility and robustness to variations in segmentation method. We discuss these reliability results along the lines of causes of modality, radiomic feature (class), and anatomical region.

Variations in radiomics measures using the different segmentation methods were lower than scan-rescan variability using any given segmentation method. Robustness in DTI consistently outperformed T1-weighted imaging, but this does not hold true for scan-rescan reproducibility. A potential cause of DTI outperforming T1-weighted imaging might be that T1-weighted images contains sharper contrast around anatomical region borders, increasing the effect of slight variations in segmentation method. Our results do therefore not present a clear preference in the robustness of one MR modality over another.

Reproducibility of shape and first-order radiomic feature classes generally outperformed higher-order feature classes. Previous work on the impact of co-registration on PET radiomics features has found similar results^56^. However, specifically on the matter of hippocampal segmentation, previous work found textural features to be the most reproducible^59^. However, single feature robustness could be application dependent, meaning that a feature that is found to be highly precise for a certain dataset and disease could have poor stability when assessed for another dataset or disease^29^. In both reproducibility and robustness, the GLSZM feature class stood out as the worst performing. On a feature level, robustness varies greatly as can be seen by the IQR in Fig. 2 and high variance in ICC in Supplementary Fig. S2.

Last, our results show that on an anatomical region level, radiomic feature reliability is relatively independent of anatomical region, apart from the CSF, choroid plexus, and inferior lateral ventricle. In these anatomically less relevant regions, low T1-intensity and low FA values are likely at the root of low reliability.

### Robustness of radiomics-based prediction

In our work, previous findings in literature and clear robustness findings allowed for manual radiomic feature selection.

We assumed that this manual selection would improve our predictive performance. Supplementary Table S1 shows that manual feature selection did outperform Chi-squared based selected features. This could be attributed to at least two factors. First due to the close resemblance between our data set and the data sets on which evidence for imaging biomarkers of sleep deprivation were found. This could have improved selection of anatomical areas, selection of relevant features as well as feature engineering. Second, in data driven feature selection we observe overfitting potentially caused by the vast amount of features per sample drowning out signal.

Due to the low performance achieved on data driven feature selection, we have decided not to go deeper into the specific features selected, as we expect them the selected sets to contain predominantly noise. In manual feature selection, expanding on these biomarkers identified has already been performed by works from which we drew our selection methods and further optimizing the sleep deprivation model lies beyond the scope of this work.

Although feature selection inherently means that some relations might have been lost and are not investigated in this work. However, model optimization and proving generalization of a predictive method on the topic of sleep deprivation is not within the scope of this work. Thus, we did not optimize hyperparameters such as model architecture or training parameters. Additionally, explanatory analysis of the contribution of radiomic features to this sleep deprivation classification lies outside of the scope of this work.

Despite the slight imbalance in the sleep deprivation labels (22/20) incurred by exclusion of subjects, accuracy still conveys the model performance in an intuitive way. Our work presents an independent comparison of methods that includes more segmentation methods than has previously been presented in literature. Radiomics prediction using deep learning based segmentation outperformed classical methods. Post hoc testing found this difference significant for FastSurfer as compared to both ASEG and SAMSEG. For Med-DeepBrain this was only significant when compared to ASEG, and not to SAMSEG. SAMSEG also significant outperformed ASEG, but not the other methods. This illustrates potential advantages of newer, specifically deep learning based methods in segmentation. Future research should confirm that these methods perform equally well or better in other applications.

### Study limitations

Main limitations of the study come down to the application sensitive nature of radiomics. First of all, with the data acquisition: our study is limited to the two modalities used and shows differences in robustness metrics. Our results regarding robustness may not necessarily generalize to other modalities such as fMRI or ASL. Second, results regarding the differences in predictive performance of different segmentations methods might not generalize to other psychoradiological applications.

The dataset was relatively homogeneous and small in size. The population consisted of relatively young healthy individuals, which improves segmentation quality and potentially lowers disagreement among segmentation methods. Due to the dataset size, the size of the test set was limited which potentially increases variation in the cross-validation performance results. Since we did not stratify for labels in our partitioning scheme, some variation could also be attributed to slight imbalances due to the small sample size of our dataset.

Whole-brain segmentation only included 32 subcortical anatomical regions. We chose to limit the scope of this research to subcortical anatomical regions for two reasons: Cortical parcellation not available for SAMSEG, and cortical labeling definitions were inconsistent between VUNO Med-DeepBrain and FreeSurfer ASEG and FastSurfer methods.

### Conclusion

Our work shows that small changes in segmentations due to a variation in image segmentation method affects radiomic features and subsequent predictive modeling when using these features. The robustness of these features is largely independent of the anatomical region, and lower-order radiomic features are generally more robust. Noteworthy, modern deep learning-based segmentation methods resulted in radiomic features that more accurately distinguishing sleep-deprived cases from controls. Our study suggests that methodological differences in fully automatic segmentation are of importance in radiomic feature-based cross-study comparison.

## Material and methods

### Dataset

A scan–rescan randomized case–control MRI neuro-imaging dataset of healthy adults (n = 46, age 26 ± 7 years; 29 women) was used, as described in previous studies^73–76^. Twenty-three subjects were randomly assigned to either a night of sleep deprivation, or a normal SWC. T1-weighted and DTI scans were acquired at four TPs over two days: TP1, around 9 a.m. after a night of normal sleep in their own home; TP2, around 8 p.m. approximately 11 h after T1; TP3 approximately 23 h after TP1; And finally, TP4, in the afternoon of the second day around 4 p.m. Participants in the normal SWC group went home to sleep between TP2 and 3, while those in the sleep deprivation group stayed at the hospital. This data collection was approved by the Regional Committee for Medical and Health Research Ethics, South-Eastern Norway (REK Sør-Øst, ref: 2017/2200) and conducted in line with the Declaration of Helsinki. All participants provided written informed consent. Data, code and documentation used in the study are available from the corresponding author upon reasonable request.

### MRI acquisition and processing

Image acquisition consisting of T1-weighted and DTI and processing and was conducted in accordance with recommended standard brain segmentation protocols provided by the FreeSurfer group^81^.

T1-weighted brain images were scanned using a 3T Siemens Magnetom Prisma scanner (Siemens Healthcare, Erlangen, Germany) using a 32-channel head coil. The acquisition parameters^81^ were as follows: repetition time (TR) = 2530 ms, echo time (TE) = 3.5 ms, flip angle = 7°. The voxel size was 1.0 × 1.0 × 1.0 mm and field-of-view (FOV) was 256 × 256 mm^2^ (256 × 256 matrix) with 176 sagittal slices. Acquisition time was six minutes three seconds.

Preprocessing of T1 data consisted of motion correction, skull stripping and intensity normalization using the FreeSurfer image preprocessing pipeline (autorecon2)^82^.

The DTI scan protocol has been described previously^73^. It consisted of a full-brain multi-shell Stejskal-Tanner pulsed mono-planar gradient scheme^83^ with a single-shot spin-echo multiband-accelerated echo-planar imaging (EPI) readout module^84^. Seventy-six axial slices with b-values = [500–1000–2000–3000–4000] (s/mm^2^) and non-coplanar diffusion-sensitized gradient directions were acquired with the corresponding numbers of gradient directions n_dir_ = [12–30–40–50–60]. The following parameters were applied: TR = 2450 ms, TE = 85 ms, flip-angle = 78°. The voxel size = 2.0 × 2.0 × 2.0 mm, FOV = 212 × 212 mm^2^ (106 × 106 matrix), slice thickness = 2 mm, and multiband acceleration factor = 4. Acquisition time was eight minutes and 21 s. In addition, five non-diffusion-weighted image sets (b = 0) of opposite phase-encode direction—but otherwise identical imaging parameters—were acquired for correction of susceptibility distortions. Acquisition time was 31 s.

Each DTI volume was affinely registered to the average non-diffusion weighted volume using the FMRIB's Linear Image Registration Tool (FLIRT)^85^, correcting for intra-scan subject motion and eddy-current distortions. After non-brain tissue was removed^86^, voxel-wise eigenvalues and eigenvectors were extracted from the estimated diffusion tensor and fractional anisotropy (FA) was calculated. The fractional anisotropy (FA) map was co-registered to the structural scan and resampled to 1.0 × 1.0 × 1.0 mm using the statistical parametric mapping (SPM) toolbox for Matlab 2019b (The MathWorks, Natick, Massachusetts). We manually checked for co-registration errors.

### Segmentation and Segmentation agreement

Four whole-brain segmentation methods were selected: FreeSurfer Automatic Segmentation (ASEG)^87^ version 7.1.0, FreeSurfer Sequence Adaptive Multimodal Segmentation (SAMSEG)^70,71^, FastSurfer^61^ and VUNO Med-DeepBrain^62^ (VUNO Inc., Seoul, South Korea) version 1.0.1. A description of each of these methods is provided in Supplementary Note S1. The selection of segmentation methods used was based on three considerations: first, the selection was limited to methods producing the same anatomical labeling. Second, methods were required to use the same modality as source, being T1-weighted MRI. And last, the different methods represent a mix of underlying methodologies. This mix consists of commonly used conventional and recently introduced methods that aim to outperform conventional methods through deep learning.

We generated segmentation labels from T1-weighted images and semantically matched these across segmentation methods, excluding areas that were not available for all methods. All segmentations were checked manually for all subjects to exclude potential failures. Segmentation agreement was determined by computing the Dice-coefficient given in equation below with X and Y being sets of segmentations. It was implemented using numpy (v. 1.23.0). For the exact implementation, we refer to the repository that can be found in the data availability statement. The Dice-coefficient was calculated for each segmentation method pair, for each anatomical area^88^. We interpreted Dice-coefficients using the same range of strength of agreement as for the Kappa coefficient and computed IQRs to aid interpretation^89^.

$$Dice\;Coefficient=\frac{2*\left|X\cap Y\right|}{\left|X\right|+\left|Y\right|}$$

### Radiomic feature extraction reliability analysis

We used seven classes to subdivide radiomic features, roughly in order of complexity: shape-based features^90^, first-order features^90^, gray level co-occurrence matrices (GLCM)^91^, gray level dependence matrices (GLDM)^92^, gray level run length matrices (GLRLM)^93^, gray level size zone matrices (GLSZM)^94^, and neighboring gray tone difference matrices (NGTDM). A description of these feature classes can be found in Supplementary Note S2.

We computed radiomic features for each anatomical area, in each scan modality and at each TP using PyRadiomics^95^ (v 0.3.0.1) implemented in Python (v. 3.8.4). Geometry tolerance was set to 10^–3^ mm. Other parameters such as bin properties were left to default and can be found in Supplementary Table S3. Feature definitions are in compliance with Imaging Biomarker Standardization Initiative (IBSI)^46^ and are described extensively at the proprietary repository^96^.

Next, we computed radiomic feature reliability for each anatomical area and each radiomic feature. Reliability was assessed for two measurements: first, scan-rescan reproducibility was calculated by computing the two-way mixed intra-class correlation (ICC)^97^ between TP1 and TP2, thus before any effects of sleep deprivation. Second, robustness to segmentation method variation was computed similarly using ICC among all four segmentation methods. ICC was implemented using the Pingouin^98^ (v. 0.3.10) for Python. Resulting values were averaged over all subjects. Throughout this work, averaging of coefficients was performed using Fisher’s z-transformation^99^. At first, computation of radiomic feature reliability produces a comprehensive overview of reliability of each feature for each MR modality. Second, reliability metrics were averaged per anatomical region and radiomic feature class. Anatomical regions with failing segmentation agreement were excluded to avoid including segmentation errors or regions with different semantical definitions that were not previously excluded in the matching of segmentation labels from affecting radiomic feature properties. Failure was defined as a Dice-coefficient below 0.5.

### Radiomics-based classification and statistical analysis

To investigate the effect of the segmentation method variation on the discriminative power of radiomic features, we used a binary classifier to separate sleep-deprived subjects from controls. This classifier was trained on the radiomic features produced by each of the four segmentation methods.

We assumed that manual feature selection outperforms data-driven feature selection for two reasons: first, the number of samples per feature is extremely low and second imaging biomarker features of sleep deprivation have been identified in previous studies^73–77^. To test this assumption, we compared the performance of manual feature selection with the 500 best Chi-squared selected features. Chi-squared feature selection was implemented in Scikit-learn^100^ (v. 1.0.2).

Manual feature selection was performed in three steps. First, a selection in modalities and radiomic features was made based on a combination of literary findings on effects of sleep deprivation and two classes with highest radiomic feature reproducibility. Second, anatomical regions with failing agreement, as described in the previous section, were excluded. Left–right hemisphere values were not averaged, such that potential asymmetric properties remained. Last, to better express the temporal relationship in the data while reducing dimensionality, the values at the four TPs were used to compute a first-order polynomial. Only the coefficient-over-time of this polynomial was used for prediction.

A neural network was trained on the remaining radiomic features. The input of the network consisted of a one-dimensional vector of radiomic features, and sleep deprivation as binary class label. The network consisted of three blocks, each consisting of a batch normalization layer, a rectified linear unit (ReLU) activation layer^101^, linear layer and a dropout regularization layer, in that order. These blocks were followed by a sigmoid layer combined with a binary cross-entropy (BCE) loss.

The network was implemented in PyTorch^102^ version 1.7.1, on a single Nvidia GeForce RTC 2080 SUPER (Nvidia Corporation, Delaware, California) graphics processing unit (GPU) with CUDA^103^ version 10.2. Adam optimization with L1-regularization, default weight initialization, and a constant learning rate of 10^–3^ were used for training and a batch size of 16. The regularization factor was set to 10^–3^ and dropout probability to 0.3. Training was performed until loss on the validation set refrained from decreasing. After training the parameters were reinstated to the state with lowest validation loss for testing.

A complete data approach, excluding subjects with missing scans, was followed. This is required to allow for the identical computation of the temporal effect using a polynomial. Data was divided into a training partition of 70%, validation partition of 20% and test partition of 10% of the included samples with which tenfold cross-validation was performed. Each fold was initialized differently, but for each method the same sequence of randomization seed was used with the same partitioning to ensure comparability of the performance of the model of each fold.

Shapiro–Wilk test was used to test normality of the paired BCE-loss performance over all folds. Friedman test implemented in SciPy^100^ version 1.6.2 was used to test the hypothesis that model performance expressed as BCE-loss did not differ among methods used. We follow this up by performing a Nemenyi post-hoc test to find which pairwise groups have significant difference. This test was implemented in the scikit-posthocs^104^ package version 0.7.0 with a significance level of 0.05.

## Supplementary Information

Supplementary Information.

## Data Availability

The authors confirm that the data supporting the findings of this study are available within the article and its supplementary material. The documented code base is available on GitHub (https://github.com/DEPREDICT/SLEEEP). Raw MRI data to support the findings of this study are available from the corresponding author, upon reasonable request.
