# Supplementary material for: Robustness of radiomics to variations in segmentation methods in multimodal brain MRI
Source: Sci Rep. 2022 Oct 6;12:16712. doi: 10.1038/s41598-022-20703-9 (PMC9537186; doi:10.1038/s41598-022-20703-9)
Supplement: Supplementary file 1 — Supplementary Information. [file 41598_2022_20703_MOESM1_ESM.docx]

# Supplement

**Supplementary Figure S1.** Examples of segmentation output


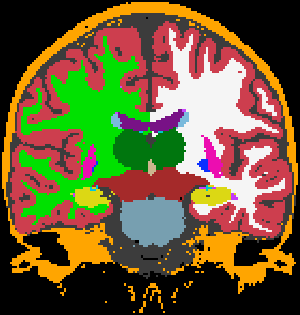

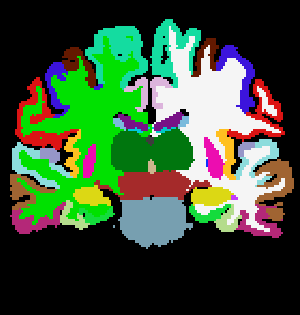

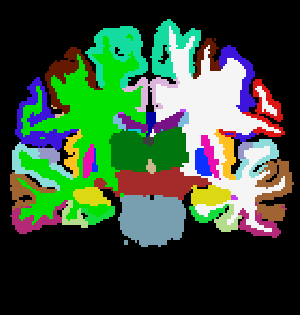

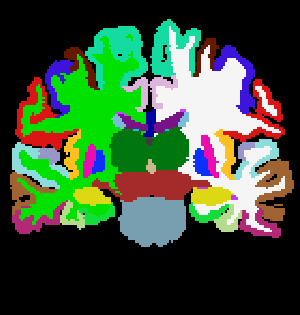


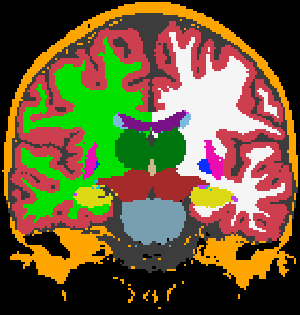

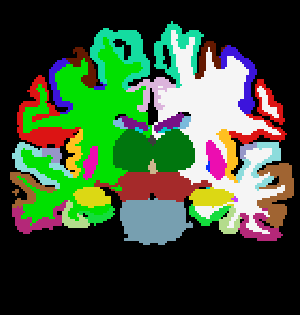

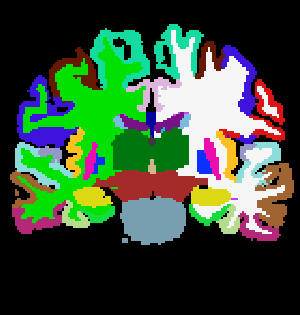

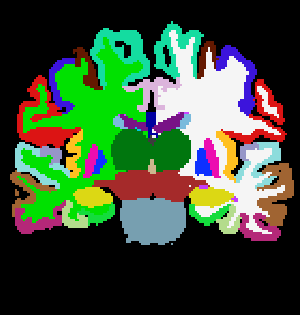


| **FreeSurfer SAMSEG** | **VUNO Med-DeepBrain** | **FreeSurfer ASEG** | **FastSurfer** |
| --- | --- | --- | --- |

Two examples of the subtlety of differences in segmentation results for four different segmentation methods best seen in color print. From left to right: SAMSEG, Med-DeepBrain, FreeSurfer ASEG and FastSurfer. Subcortical differences are best visible lateral to the thalamus at the putamen (pink) and pallidum (blue), or cranial to the thalamus at the caudate nucleus (cyan) under the lateral ventricle (purple).

The yellow arrow points at label color for CSF. Only SAMSEG segments CSF surrounding the cortex, resulting in the low dice pair agreement seen earlier (Fig. 1).

**Supplementary Figure S2**. An extensive overview of the robustness of radiomic features.


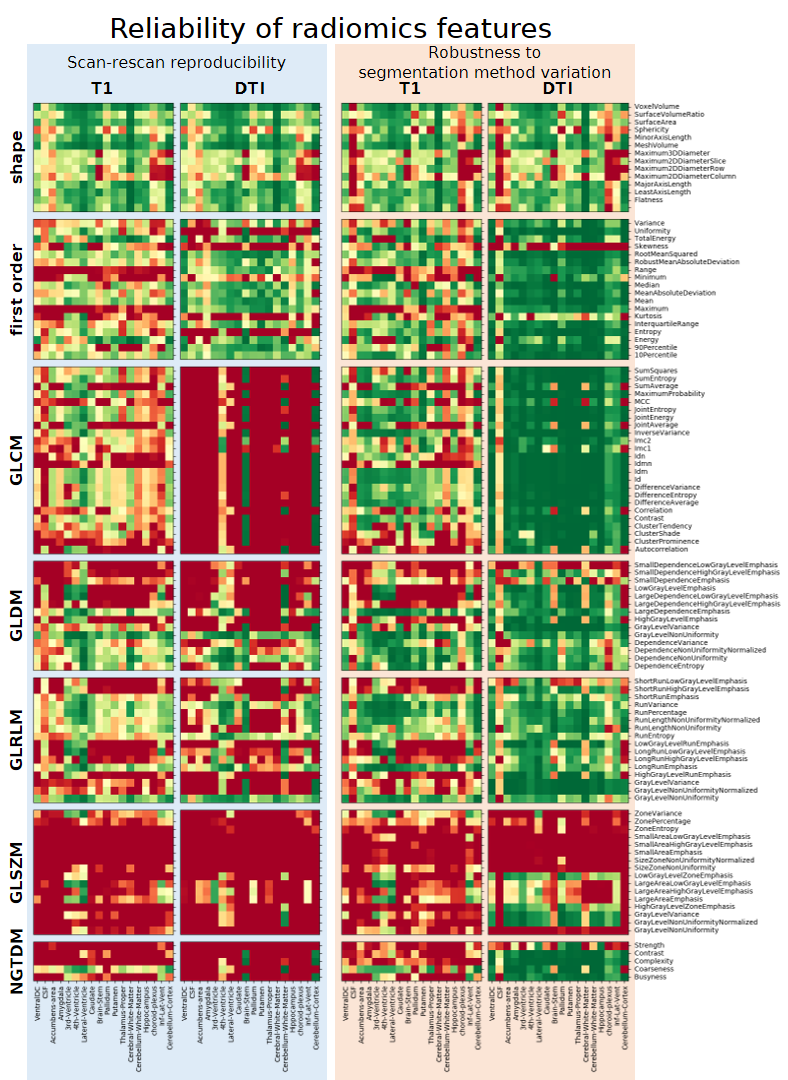


Radiomic feature reliability measured using ICC. The left two columns show scan-rescan reproducibility. The right two show robustness to segmentation method variation. The color scale runs from red (worse) through yellow to green (better) with limits [0.5, 1].

The vertical axis is comprised of 107 radiomic features, categorized by feature class shown on the left side of the chart. Abbreviations of feature classes can be found in **Supplementary Note S6***.* Vertical bar-patterns can be interpreted as anatomical-specific robustness deviation. A clear example of under-performing regions are the choroid plexus and inferior lateral ventricle in DTI segmentation method robust-ness.

On the horizontal axis of each column the same eighteen anatomical labels are showed as used in Figure 1.Horizontal bar-patterns can be interpreted as radiomic feature-specific robustness deviation. Take for example NGTDM-coarseness, scoring better than other NGTDM-features across the board.

The bar chart of Figure 2 shows an aggregation of each block of each column.

**Supplementary Table S3.** Classification performance of pipelines for each segmentation method using Chi-Squared feature selection (CHI-SQUARED) and manual feature selection (ORIGINAL). Median performance of cross-validation folds and IQR are shown. Coloring runs from red (worst) through yellow (50-th percentile) to green (best). Two color ranges are used: one for the loss (lower is better) and one for the accuracy (higher is better) columns.

|  | **BCE LOSS** | **BCE LOSS** | **ACCURACY** | **ACCURACY** |
| --- | --- | --- | --- | --- |
|  | **(CHI-SQUARED)** | **(ORIGINAL)** | **(CHI-SQUARED)** | **(ORIGINAL)** |
| **SAMSEG** | 0.68 (0.60-0.74) | 0.53 (0.37-0.70) | 57% (57%-67%) | 77% (60%-89%) |
| **Med-DeepBrain** | 0.72 (0.68-0.75) | 0.50 (0.30-0.69) | 53% (43%-63%) | 82% (62%-91%) |
| **ASEG** | 1.04 (0.98-1.09) | 0.60 (0.47-0.72) | 55% (45%-65%) | 71% (62%-82%) |
| **FastSurfer** | 0.69 (0.64-0.74) | 0.46 (0.25-0.66) | 56% (39%-74%) | 84% (74%-92%) |

**Supplementary Table S4.** Pairwise results for Nemenyi post-hoc test. Significant differences (α = 0.05) are denoted with an asterisk.

|  | **SAMSEG** | **MED-DEEPBRAIN** | **ASEG** | **FASTSURFER** |
| --- | --- | --- | --- | --- |
| **SAMSEG** | 1.000 | 0.177 | 0.027* | 0.027* |
| **Med-DeepBrain** | 0.177 | 1.000 | 0.001* | 0.858 |
| **ASEG** | 0.027* | 0.001* | 1.000 | 0.001* |
| **FastSurfer** | 0.027* | 0.858 | 0.001* | 1.000 |

**Supplementary Note S5.** Description of segmentation methods

- ASEG is the most extensively used whole-brain segmentation method. ASEG uses a probabilistic-based atlas which captures information about the location of structures to determine the relationship between imagine intensity and neuroanatomical labels.^1^ This type of atlas makes use of information regarding the statistical properties of anatomical structures to store the image a space in which coordinates have anatomical meaning as opposed to the somewhat arbitrary coordinates in a raw image. It is distributed via the FreeSurfer analysis package.^2^ In this work, version 7.1.0 was used.
- SAMSEG is a recently introduced iteration on ASEGs atlas-based approach. SAMSEG uses generative parametric models which encodes probabilistic atlases using Bayesian interference.^3^ SAMSEG is less computationally demanding and has shown improvement in robustness and sensitivity over FreeSurfer.^1^

The remaining two methods are deep learning-based and have been recently introduced.

- FastSurfer was introduced in 2020 and is based on a DenseNet architecture.^4^
- VUNO Med-DeepBrain is based on work by Suh et al., published in 2019. Their architecture is HighResNet-based.^5^

Both demonstrate high robustness on a scan-rescan dataset, and sensitivity on an Alzheimer’s disease dataset. Only FastSurfer shows direct comparison to FreeSurfer as alternative method.

**Supplementary Note S6.** Description of radiomic feature classes

- Shape-based features,^6,7^ also known as semantic features include volume, elongation and surface area.
- First-order features are often histogram-based and examine gray level signal intensity. Examples of these features include uniformity, entropy, mean, median and kurtosis.^6,8^
- Second-order features can be thought of as “texture” features. These examine spatial relationship between gray level signal intensities by constructing a gray-level dependence matrix^6,8^ Three examples of types of features are:
  - Gray level co-occurrence matrix (GLCM) which analyzes occurrence of different gray level voxel pairs in different directions at a predetermined distance.^9^
  - Gray level dependence matrix (GLDM), with dependency defined as a ^10^
  - Gray level run length matrix (GLRLM), which quantifies the number of consecutive voxels with same gray level. ^11^
  - Gray level size zone matrix (GLSZM), which quantifies the size of a homogenous area of an image.^12^
  - Neighboring gray tone difference matrix (NGTDM), which quantifies the difference between a gray value and the average gray value of its neighbors.
- Higher-order features involve application of a filter or transformation to an image prior to feature extraction. These features aim to identify patterns or highlight details within the image that are hard to interpret and do not have a standardized definition.^13^

**Supplementary Table S7.** Parameters used for PyRadiomics (v.3.0.1) feature extraction. Non default parameter is marked in yellow.

| **Feature Extractor**  *Image Normalization* | |  | **Filter**  *Wavelet settings* | |  | **Feature Class Specific** | |
| --- | --- | --- | --- | --- | --- | --- | --- |
| normalize | False |  | sigma | [] |  | voxelArrayShift | 0 |
| normalizeScale | 1 |  | start_level | 0 |  | symmetricalGLCM | True |
| removeOutliers | None |  | level | 1 |  | gldm_a | 0 |
| *Resampling the image* | |  | *Gradient settings* | |  | wavelet | coif1 |
| resampledPixelSpc | None |  | gradientUseSpc | True |  | kernelRadius | 1 |
| interpolator | sitkBSpline |  | lbp2DRadius | 1 |  | maskedKernel | True |
| padDistance | 5 |  | lbp2DSamples | 9 |  | initValue | 0 |
| *Pre-Cropping* |  |  | lbp2DMethod | uniform |  | voxelBatch | -1 |
| preCrop | False |  |  |  |  |  |  |
| *Resegmentation* |  |  | **Feature Class** |  |  |  |  |
| resegmentRange | None |  | Label | 1 |  |  |  |
| resegmentMode | solute |  | weightingNorm | None |  |  |  |
| *Mask validation* |  |  | distances | [1] |  |  |  |
| minimumROIDims | 2 |  | *Forced 2D extraction* | |  |  |  |
| minimumROISize | None |  | force2D | False |  |  |  |
| geometryTolerance | 10-3 |  | force2Ddims | 0 |  |  |  |
| correctMask | False |  | *Image discretization* | |  |  |  |
| *Miscellaneous* |  |  | binWidth | 25 |  |  |  |
| additionalInfo | True |  | binCount | None |  |  |  |
|  |  |  |  |  |  |  |  |
|  |  |  |  |  |  |  |  |
|  |  |  |  |  |  |  |  |
|  |  |  |  |  |  |  |  |
|  |  |  |  |  |  |  |  |
|  |  |  |  |  |  |  |  |
